# Comparison of long-term efficacy, tolerability and drug survival rates of baricitinib as monotherapy or combination therapy in RA patients: data from a real-world prospective cohort study

**DOI:** 10.1136/rmdopen-2025-006333

**Published:** 2025-12-17

**Authors:** Alp Temiz, Koray Tascilar, Arnd Kleyer, Axel J Hueber, Fabian Hartmann, Georg Schett, Sara Bayat

**Affiliations:** 1 Department of Medicine 3 - Rheumatology and Immunology, Friedrich-Alexander-Universität Erlangen-Nürnberg and Universitätsklinikum Erlangen, Erlangen, Germany; 2 Deutsches Zentrum Immuntherapie (DZI), Friedrich-Alexander-Universität Erlangen-Nürnberg and Universitätsklinikum Erlangen, Erlangen, Germany; 3 Department of Rheumatology and Clinical Immunology, Charité - Universitätsmedizin Berlin, Berlin, Germany; 4 Division of Rheumatology, Klinikum Nürnberg, Paracelsus Medical University, Nürnberg, Germany

**Keywords:** Arthritis, Rheumatoid, DMARD, Epidemiology

## Abstract

**Background:**

Janus kinase inhibitors (JAKi), like baricitinib, are used as monotherapy (MONO) and in combination with methotrexate (MTX) in rheumatoid arthritis (RA) patients. This study aimed to evaluate the long-term efficacy, drug survival and safety of baricitinib MONO versus combination therapy (COMBO) in a prospective cohort of patients with RA.

**Methods:**

We analysed data from 219 RA patients initiating baricitinib in a single-centre, prospective observational cohort between 2017 and 2023. Clinical data, patient-reported outcomes and safety events were collected every 3 months. Drug survival was analysed using the Kaplan-Meier method, and longitudinal outcomes were assessed using linear mixed-effects models.

**Results:**

The cohort included 219 patients (165 MONO, 54 COMBO) with high baseline disease activity (mean DAS28-ESR 4.0). Both groups showed rapid and sustained clinical improvement, with disease activity stabilising in the low range by 6 months of treatment. One third of the patients (n=72, 33%) achieved Boolean remission, with no significant difference between groups (p=0.35). Median drug survival was 36 months and was similar for both regimens (log-rank p=0.82). Discontinuation due to adverse events occurred in 10.9% of patients. Four thrombotic events were noted, all in patients with pre-existing cardiovascular risk factors, with no new safety signals emerging.

**Conclusions:**

In this real-world cohort, baricitinib demonstrated sustained long-term effectiveness, drug survival and an acceptable safety profile. The similar outcomes observed for MONO and COMBO indicate that baricitinib may be used effectively as MONO in patients with RA for whom MTX is not suitable or tolerated.

**WHAT IS ALREADY KNOWN ON THIS TOPIC**

- Baricitinib has shown efficacy in patients in rheumatoid arthritis (RA) across multiple controlled trials.
- In the treatment of RA, baricitinib is used as monotherapy (MONO) or in combination with methotrexate and other conventional synthetic disease-modifying antirheumatic drugs.
- Prospective observational real-world studies that assess the efficacy and safety of baricitinib as MONO or combination therapy (COMBO) are scarce.

**WHAT THIS STUDY ADDS**

- In this 6 years prospective real-world cohort of patients with RA, baricitinib yielded sustained control of inflammation and improvement of function and quality of life as MONO or COMBO.
- Breadth of clinical responses, remission rates, drug survival rates and tolerability were similar among baricitinib MONO or COMBO.
- Treatment with baricitinib was safe as MONO or COMBO over 6 years follow-up.

**HOW THIS STUDY MIGHT AFFECT RESEARCH, PRACTICE OR POLICY**

- This study stimulates the use of baricitinib as MONO and underlines the long-term safety of treatment with baricitinib in patients with RA.

## Introduction

 Rheumatoid arthritis (RA) is a chronic inflammatory disease of the joints requiring sustained control of inflammation by cytokine-inhibiting drugs.^1^ Janus kinase inhibitors (JAKi), a class of targeted synthetic disease-modifying antirheumatic drugs (tsDMARDs), are highly effective to control inflammation in RA. However, their safety profile has been debated due to conflicting data on the risk of major adverse cardiovascular events, malignancies and thromboembolic events.^2^,^4^ This led to recent EULAR recommendations advising careful consideration of JAKi in RA patients with a high cardiovascular risk.^5^ Nevertheless, JAKi remain an important asset in the treatment of RA, particularly in patients who cannot use conventional synthetic DMARDs (csDMARDs).^5 6^

Baricitinib is an effective JAK inhibitor for the treatment of RA and may offer additional benefits such as improved bone quality.^7^,^11^ Pivotal Phase III trials in bDMARD-inadequate responders,^12^ csDMARD-inadequate responders^13^ and methotrexate-inadequate responders^7^ established its efficacy. Long-term data from Phase III trials showed sustained efficacy of baricitinib for up to 6.5 years, but these studies lacked detailed reporting on safety parameters.^14^ In both randomised controlled trials (RCTs) and routine clinical practice, baricitinib has shown comparable efficacy as a monotherapy (MONO) and in combination with methotrexate (MTX).^15 16^ However, a meta-analysis of RCTs suggested that while combination therapy (COMBA) is superior in efficacy, it may carry a higher risk of adverse events (AEs) leading to discontinuation.^17^

These findings from RCTs may not fully translate to daily clinical practice, where patients are typically older, have a longer disease duration and more comorbidities. A recent analysis of observational data by Edwards *et al*, which included our own Erlangen cohort, demonstrated the effectiveness of baricitinib MONO over 6 to 12 months.^16 18^ Building on these findings, this study aims to assess the long-term efficacy and safety of baricitinib in a larger patient population from the Erlangen cohort with an extended follow-up period, directly comparing outcomes between MONO and COMBO.

## Methods

### Patients and assessments

In this single-centre, prospective observational cohort study (2017–2023), we enrolled patients with RA (per 2010 ACR/EULAR criteria) who were initiating treatment with baricitinib 4 mg one daily. All patients received baricitinib through standard clinical care and on-label prescription according to national treatment guidelines. The observation started at the time of the first prescription. In Germany, baricitinib is reimbursed by the statutory health insurance, and patients did not have to cover any medication costs themselves. No study medication was provided by a pharmaceutical company. Details of patient enrolment and assessments have been described previously.^16^

The study was approved by the local ethics committee (ref: 19_18 B), and all patients provided written informed consent. At baseline and at 3-month intervals, we systematically collected patient demographics, comorbidities, laboratory parameters including C-reactive protein, erythrocyte sedimentation rate (ESR), rheumatoid factor (RF) and anti-cyclic citrullinated peptide (CCP) antibodies were recorded. Reasons for treatment discontinuation were assessed. Disease activity was measured via disease activity score (DAS) 28, simplified disease activity index (SDAI) and fulfilment of Boolean remission criteria. Patient-reported outcomes included the HAQ-DI, RAID and FACIT instruments.

### Statistical analysis

Longitudinal outcomes were analysed using linear mixed-effects models to calculate estimated marginal means. Drug survival and time to remission were assessed using the Kaplan-Meier method, with group differences compared by the log-rank test. A p value <0.05 was considered statistically significant. All analyses were performed in R (V.4.5.1) using the lmerTest, emmeans and survival packages.

## Results

### Patient characteristics

A total of 219 patients treated with baricitinib between 2017 and August 2023 were analysed (157 women, 62 men; mean age 59.8±12.6 years; disease duration 8.8±10.0 years). Of these, 165 received baricitinib as a MONO and 54 as COMBO. Baseline DAS28-ESR and SDAI were 4.0±1.1 and 20.0±9.8, respectively, with HAQ-DI, RAID and FACIT scores of 1.1±0.6, 4.7±2.2 and 31.0±12.0. Eighty-three patients (37.9%) were b/tsDMARD-naïve, more frequently in the COMBO group (64.8%) than the MONO group (29.1%). Other baseline characteristics were largely comparable between groups (table 1).

**Table 1:** Baseline characteristics of patients treated with baricitinib monotherapy or in combination with methotrexate

Variable	Overall	Combination	Monotherapy
N	219	54	165
Sex (F/M)	157/62	32/22	125/40
ACPA positive (N (%))	142 (64.8)	39 (72.2)	103 (62.2)
DAS28-ESR	4.1 (0.9)	4.0 (1.2)	4.0 (1.2)
SDAI	22.4 (9.7)	19.3 (9.9)	19.3 (9.9)
ESR (mm/hour)	20.1 (20.7)	20.9 (16.8)	20.9 (16.8)
CRP (mg/L)	12.3 (10.5)	12.3 (17.9)	12.3 (17.9)
TJC28	4.6 (4.4)	4.6 (4.9)	4.6 (4.9)
SJC28	4.0 (4.5)	3.4 (4.3)	3.4 (4.3)
TJC78	5.6 (6.5)	5.8 (5.6)	5.8 (5.6)
SJC76	4.4 (6.8)	4.0 (4.5)	4.0 (4.5)
VAS—Disease Activity (patient)	52.0 (17.0)	49.1 (23.8)	49.1 (23.8)
VAS—Disease Activity (physician)	54.9 (16.6)	46.6 (22.7)	46.6 (22.7)
VAS—Pain	57.5 (20.1)	50.2 (26.3)	50.2 (26.3)
Smoking (pack-years)	30.0 (21.5)	35.2 (46.5)	35.2 (46.5)
FACIT score	32.0 (11.2)	30.7 (12.4)	30.7 (12.4)
HAQ-DI	1.1 (0.6)	1.1 (0.7)	1.1 (0.7)
RAID	4.7 (2.0)	4.7 (2.2)	4.7 (2.2)
Morning stiffness (min)	61.8 (64.2)	40.9 (45.9)	40.9 (45.9)

Values are presented as mean (SD) unless otherwise indicated. Combination: baricitinib plus methotrexate; Monotherapy: baricitinib alone.

ACPA, anti-citrullinated protein antibody; CRP, C-reactive protein; DAS28-ESR, Disease Activity Score 28 joints with erythrocyte sedimentation rate; ESR, erythrocyte sedimentation rate; FACIT, Functional Assessment of Chronic Illness Therapy; HAQ-DI, Health Assessment Questionnaire–Disability Index; RAID, Rheumatoid Arthritis Impact of Disease; SDAI, Simplified Disease Activity Index; SJC, swollen joint count; TJC, tender joint count; VAS, visual analogue scale.

### Drug survival and adverse events

**Figure 1:**
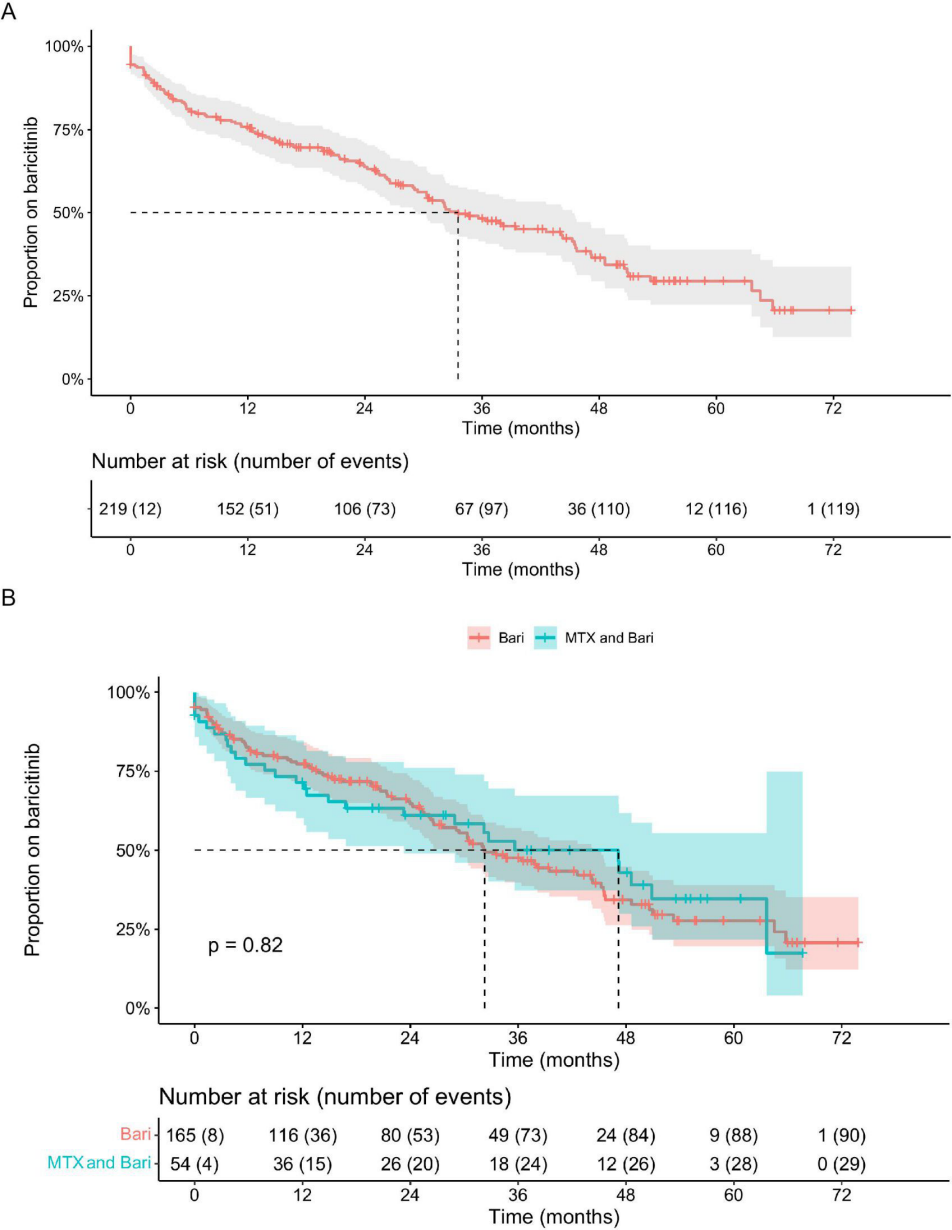
Drug survival of baricitinib monotherapy versus combination therapy with methotrexate. (A) Kaplan–Meier curves showing drug survival of baricitinib monotherapy (MONO, red) and baricitinib in combination with methotrexate (COMBO, blue) over 72 months. Shaded areas represent 95% CIs. The log-rank p value and median drug survival are indicated. (**B**) Causes for baricitinib discontinuation stratified by treatment group. Discontinuation was attributed to adverse events, lack of efficacy, remission or other reasons (eg, patient preference or cardiovascular risk reassessment). The number of patients at risk is displayed below the Kaplan–Meier curves. MTX, methotrexate.

Drug survival over 72 months was similar between MONO and COMBO groups (figure 1A, log-rank p=0.82), with a median survival of 36 months. Discontinuations occurred due to lack of efficacy (23.7%), AEs (10.9%), remission (5.0%), or other reasons such as patient preference or cardiovascular risk reassessment (16.4%) (figure 1B). Dose reduction from 4 mg to 2 mg was documented in 15 patients, primarily due to sustained remission (n=12).

AEs leading to discontinuation (n=24) were recorded in 16 patients (9.7%) in the MONO group and 8 patients (14.8%) in the COMBO group. These included recurrent infections (n=7), thrombosis (n=4), elevated creatine kinase (n=3), transaminase elevation (n=3), gastrointestinal complaints (n=3), insomnia (n=2), weight gain (n=2), thrombocytosis (n=1) and exanthema (n=1), with some patients experiencing more than one AE. All four thrombosis events were associated with pre-existing risk factors. No new cases of cardiovascular disease or malignancy were observed during follow-up.

### Clinical and serological outcomes

**Figure 2:**
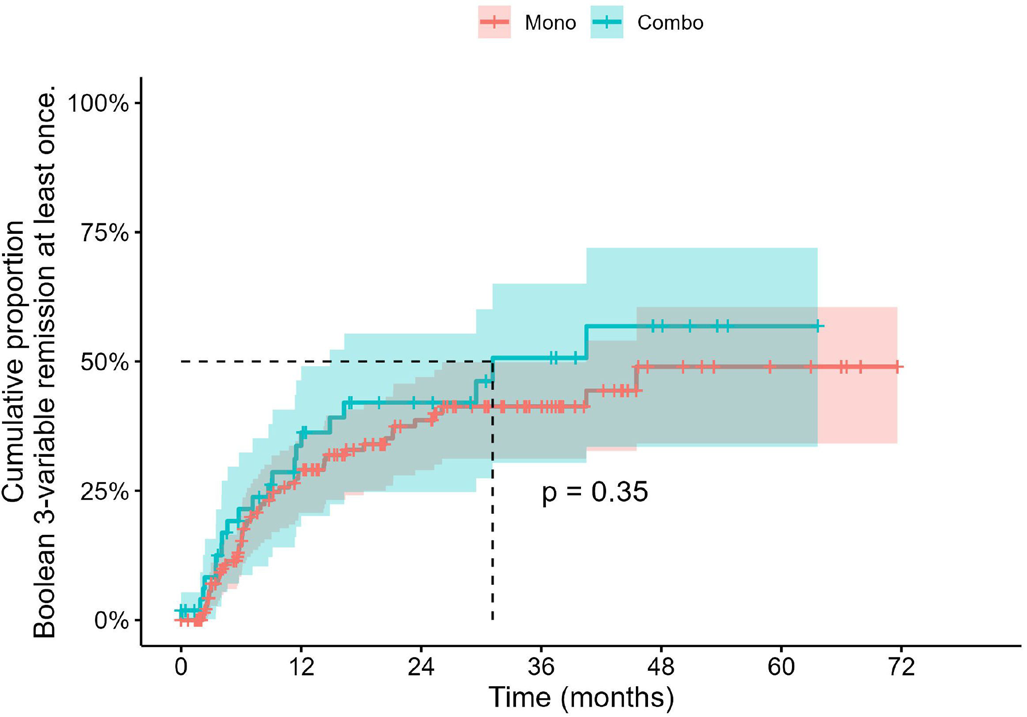
Cumulative probability of achieving Boolean 3-variable remission with baricitinib monotherapy and combination therapy. Kaplan–Meier curves display the cumulative proportion of patients who achieved Boolean 3-variable remission at least once over 72 months for baricitinib monotherapy (Mono, red) and combination therapy with methotrexate (Combo, blue). Shaded areas represent 95% CIs. The median time to first remission (dashed lines) and log-rank p value are indicated. Below the plot, the number of patients at risk and the number of remission events at each time point are presented in parentheses.

Boolean remission was achieved by 72 patients (33%), with no significant difference between MONO and COMBO groups (figure 2, p=0.35). Longitudinal analyses showed both MONO and COMBO groups experienced rapid improvements from baseline across most clinical disease activity measures within the first 6 months (online supplemental figure 1; details in online supplemental table 1). For composite scores like DAS28-ESR, the COMBO group achieved and maintained numerically lower mean scores, ending at a mean of 1.93 (95% CI 1.09 to 2.78) at Month 48, compared with 2.72 (95% CI 2.28 to 3.15) for the MONO group. Patient-reported outcomes also improved in both arms, with the COMBO group showing more pronounced and sustained improvements in HAQ-DI and FACIT scores. A significant distinction was observed in serological markers; hence, the levels of RF and anti-CCP antibodies progressively declined in the COMBO group, whereas in the MONO group, anti-CCP antibody levels showed a modest decrease, while RF levels even slightly increased. Body mass index remained stable in both groups. It is noteworthy that the 95% CIs for many estimates, particularly in the COMBO group, widened considerably at later time points, suggesting greater variability.

## Discussion

This long-term analysis provides valuable data on the effects of baricitinib over a 6 year period in routine clinical practice. Efficacy of baricitinib MONO was very similar to COMBO and also drug survival has been indistinguishable among the groups. One-third of patients achieved Boolean 3-variable remission, with comparable response rates in the MONO and COMBO groups. This efficacy data are in accordance with other cohorts, such as RA-BE-REAL,^9 11^ ORBIT-RA,^19^ SUSTAIN,^18^ BSRBR-RA,^20^ SCQM-RA,^21^ showing rapid improvement of disease activity.

Notably, in our cohort, we did not observe meaningful differences between baricitinib MONO and COMBO. Decreases in DAS28 and SDAI scores, remission rates and drug survival were similar. Numerically, COMBO yielded slightly better results; however, the differences were minimal and not statistically significant. AEs were somewhat more frequent in the combination group. These findings suggest that baricitinib MONO may represent a reasonable and effective option in clinical practice, particularly in patients with intolerance or contraindications to MTX, as previously reported.^16^ However, our observational methodology does not allow a firm recommendation against COMBO, and treatment choice should remain individualised.

Regarding safety, discontinuation of baricitinib was observed in 11% of patients due to AEs and in 24% due to a lack of efficacy. Treatment was discontinued in four patients due to thrombotic events, two of which were deep vein thrombosis. The other events were atypical (portal vein thrombosis, central retinal vein thrombosis). All patients had individual risk factors. Nevertheless, therapy was stopped due to the known increased thromboembolic risk associated with JAK inhibitors. Importantly, no new cardiovascular events or malignancies were reported during our extended follow-up. This finding supports data from other registries that baricitinib has an acceptable safety profile. These observations are particularly relevant given the concerns raised by the ORAL Surveillance trial, reinforcing the importance of individualised risk stratification. Consistent with prior reports from SUSTAIN and BSRBR-RA, our analyses also showed good tolerability when the baricitinib dose was reduced to 2 mg/day due to sustained remission or safety considerations. This observation supports the flexible, individualised use of baricitinib in the long-term care.

In conclusion, these long-term data provide strong evidence that baricitinib maintains its clinical efficacy, drug survival and acceptable safety profile as MONO over several years in a real-world setting.

## Supplementary material

10.1136/rmdopen-2025-006333 online supplemental file 1

## Data Availability

Data from this study can be requested from the corresponding author upon reasonable request.
