# Supplementary material for: Comparison of long-term efficacy, tolerability and drug survival rates of baricitinib as monotherapy or combination therapy in RA patients: data from a real-world prospective cohort study
Source: RMD Open. 2025 Dec 17;11(4):e006333. doi: 10.1136/rmdopen-2025-006333 (PMC12716592; doi:10.1136/rmdopen-2025-006333)
Supplement: online supplemental file 1 [file rmdopen-11-4-s001.docx]

**SUPPLEMENTARY MATERIAL**

**
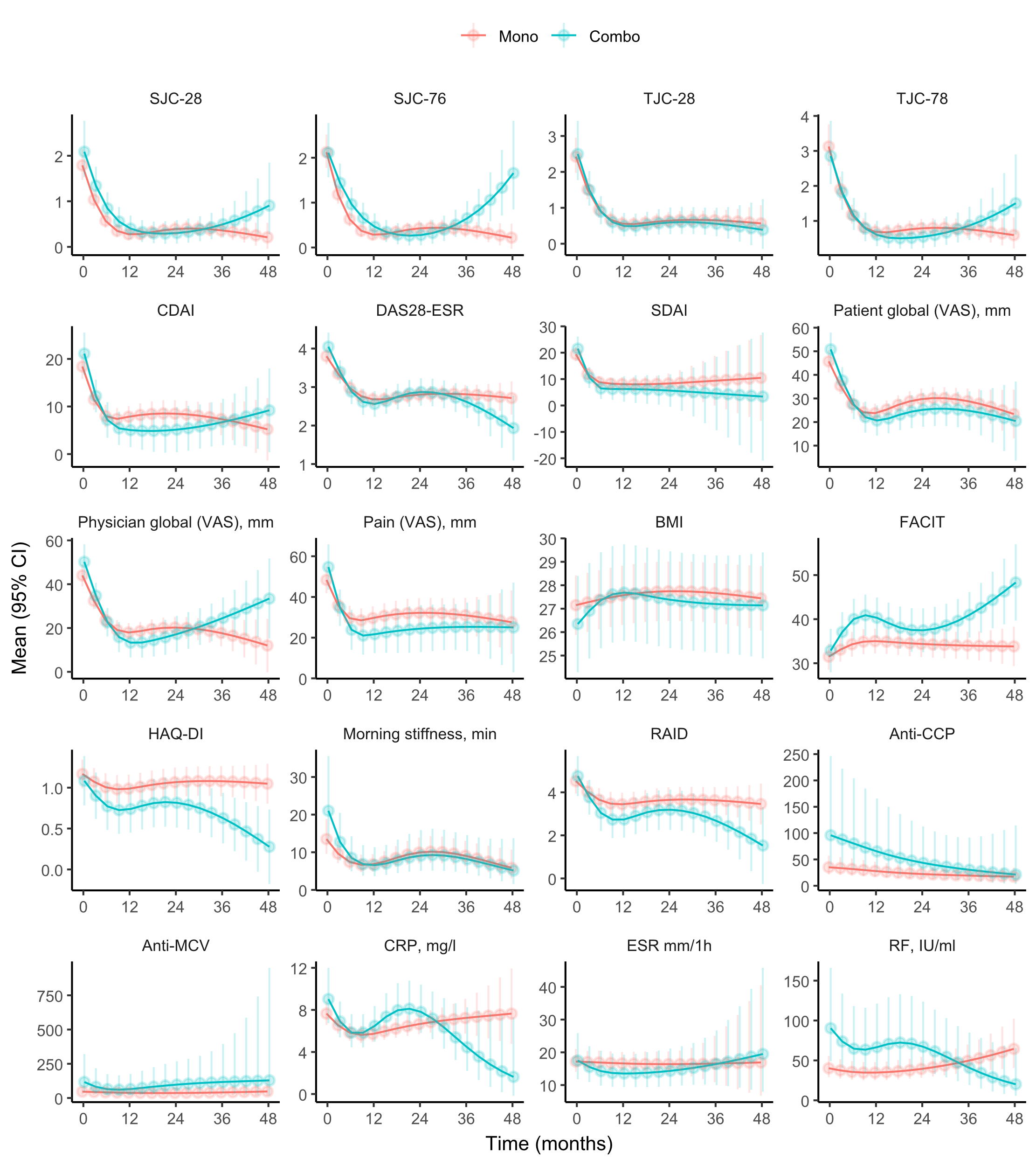
**

**Supplementary Figure 1.**

Longitudinal outcomes over 48 months for patients on baricitinib monotherapy (red) versus baricitinib plus MTX combination therapy (blue), presented as means with 95% confidence intervals. Both groups demonstrated rapid improvements in disease activity and inflammatory markers, which then stabilized. Combination therapy showed a trend toward greater improvement, though confidence intervals frequently overlapped. Autoantibody levels and BMI remained stable throughout the follow-up.

**Table 1. Longitudinal Clinical and Laboratory Outcomes for Patients on Baricitinib Monotherapy versus Combination Therapy.**

| **Variable** | **Therapy** | **Month 0** | **Month 12** | **Month 24** | **Month 36** | **Month 48** |
| --- | --- | --- | --- | --- | --- | --- |
| **SJC-28** | Mono | 1.79 (1–2) | 0.28 (0–0) | 0.39 (0–1) | 0.36 (0–1) | 0.21 (0–1) |
|  | Combo | 2.09 (2–3) | 0.4 (0–1) | 0.3 (0–1) | 0.5 (0–1) | 0.91 (0–2) |
| **SJC-76** | Mono | 2.12 (2–3) | 0.29 (0–0) | 0.43 (0–1) | 0.4 (0–1) | 0.22 (0–1) |
|  | Combo | 2.11 (2–3) | 0.46 (0–1) | 0.28 (0–1) | 0.65 (0–1) | 1.66 (1–3) |
| **TJC-28** | Mono | 2.43 (2–3) | 0.56 (0–1) | 0.64 (0–1) | 0.65 (0–1) | 0.57 (0–1) |
|  | Combo | 2.5 (2–3) | 0.49 (0–1) | 0.59 (0–1) | 0.56 (0–1) | 0.39 (0–1) |
| **TJC-78** | Mono | 3.13 (3–4) | 0.69 (0–1) | 0.79 (1–1) | 0.76 (1–1) | 0.59 (0–1) |
|  | Combo | 2.85 (2–4) | 0.6 (0–1) | 0.55 (0–1) | 0.87 (0–1) | 1.5 (1–3) |
| **CDAI** | Mono | 18.4 (16–21) | 7.85 (6–10) | 8.5 (6–11) | 7.36 (4–11) | 5.22 (0–12) |
|  | Combo | 21.12 (17–26) | 5.04 (1–9) | 5.15 (0–10) | 6.75 (2–12) | 9.2 (0–18) |
| **DAS28-ESR** | Mono | 3.8 (4–4) | 2.68 (2–3) | 2.8 (3–3) | 2.81 (3–3) | 2.72 (2–3) |
|  | Combo | 4.05 (4–4) | 2.56 (2–3) | 2.87 (3–3) | 2.61 (2–3) | 1.93 (1–3) |
| **SDAI** | Mono | 19.4 (17–22) | 8.1 (6–11) | 8.34 (5–12) | 9.38 (0–19) | 10.47 (0–27) |
|  | Combo | 21.54 (17–26) | 6.26 (1–11) | 5.65 (0–12) | 4.54 (0–18) | 3.4 (0–28) |
| **Patient global (VAS)** | Mono | 45.71 (41–50) | 23.77 (20–28) | 29.71 (25–34) | 28.88 (24–34) | 23.38 (13–34) |
|  | Combo | 50.81 (44–58) | 20.64 (14–27) | 25.23 (18–32) | 24.73 (16–33) | 20.44 (4–37) |
| **Physician global (VAS)** | Mono | 44.01 (39–49) | 17.96 (14–22) | 20.21 (15–25) | 17.71 (12–24) | 12.02 (0–24) |
|  | Combo | 50.21 (42–58) | 13.32 (7–20) | 17.23 (9–26) | 24.6 (15–34) | 33.44 (15–52) |
| **Pain (VAS, min)** | Mono | 48.36 (42–55) | 29.64 (24–35) | 32.21 (25–39) | 30.99 (22–39) | 27.6 (12–43) |
|  | Combo | 54.71 (44–66) | 21.69 (12–31) | 24.46 (13–36) | 25.3 (13–37) | 25.05 (3–47) |
| **BMI** | Mono | 27.15 (26–28) | 27.57 (26–29) | 27.74 (26–29) | 27.67 (26–29) | 27.44 (26–29) |
|  | Combo | 26.35 (24–28) | 27.69 (26–30) | 27.38 (25–29) | 27.2 (25–29) | 27.14 (25–29) |
| **FACIT** | Mono | 31.5 (29–34) | 35.02 (32–38) | 34.39 (31–37) | 34.01 (31–37) | 33.83 (29–38) |
|  | Combo | 32.87 (28–38) | 40.31 (36–45) | 37.48 (32–42) | 40.98 (36–46) | 48.33 (40–57) |
| **HAQ-DI** | Mono | 1.17 (1–1) | 0.99 (1–1) | 1.07 (1–1) | 1.08 (1–1) | 1.05 (1–1) |
|  | Combo | 1.08 (1–1) | 0.74 (0–1) | 0.82 (1–1) | 0.63 (0–1) | 0.28 (0–1) |
| **Morning stiffness, min** | Mono | 13.5 (10–19) | 6.81 (5–9) | 9.93 (7–14) | 9.07 (6–13) | 5.87 (3–11) |
|  | Combo | 21.03 (12–36) | 6.57 (4–11) | 9.11 (5–16) | 8.16 (4–15) | 5.15 (2–14) |
| **RAID** | Mono | 4.52 (4–5) | 3.44 (3–4) | 3.65 (3–4) | 3.63 (3–4) | 3.46 (3–4) |
|  | Combo | 4.75 (4–6) | 2.74 (2–4) | 3.19 (2–4) | 2.67 (2–4) | 1.53 (0–3) |
| **Anti-CCP** | Mono | 35.13 (21–57) | 28.02 (17–46) | 22.37 (13–37) | 19.42 (11–33) | 17.39 (9–34) |
|  | Combo | 95.9 (37–246) | 65.23 (25–166) | 43.42 (16–113) | 30.26 (10–92) | 21.4 (3–115) |
| **Anti-MCV** | Mono | 46.36 (27–78) | 37.81 (22–63) | 36.35 (21–61) | 40.52 (21–77) | 47.65 (16–136) |
|  | Combo | 116.61 (42–321) | 65.18 (24–176) | 97.72 (35–268) | 117.07 (34–395) | 128.7 (17–951) |
| **CRP, mg/l** | Mono | 7.65 (7–9) | 5.71 (5–7) | 6.65 (6–8) | 7.23 (6–9) | 7.65 (5–12) |
|  | Combo | 9.03 (7–12) | 6.48 (5–8) | 7.81 (6–10) | 4.44 (2–8) | 1.63 (0–7) |
| **ESR mm/1h** | Mono | 17.2 (14–21) | 16.62 (14–20) | 16.4 (13–20) | 16.57 (11–25) | 16.91 (7–40) |
|  | Combo | 17.5 (12–26) | 13.53 (9–19) | 14.34 (10–21) | 16.45 (10–27) | 19.49 (8–46) |
| **RF, IU/ml** | Mono | 40.19 (29–56) | 34.76 (25–48) | 39.32 (28–54) | 49.42 (35–70) | 64.46 (40–102) |
|  | Combo | 90.38 (49–166) | 66.62 (36–121) | 67.1 (36–124) | 40.62 (19–85) | 20.11 (6–65) |

Data are presented as estimated marginal means (95% confidence interval), derived from linear mixed-effects models. SJC, Swollen Joint Count; TJC, Tender Joint Count; CDAI, Clinical Disease Activity Index; DAS28-ESR, Disease Activity Score 28 using Erythrocyte Sedimentation Rate; SDAI, Simplified Disease Activity Index; VAS, Visual Analogue Scale (min); BMI, Body Mass Index; FACIT, Functional Assessment of Chronic Illness Therapy - Fatigue; HAQ-DI, Health Assessment Questionnaire-Disability Index; RAID, Rheumatoid Arthritis Impact of Disease; Anti-CCP, Anti-Cyclic Citrullinated Peptide; Anti-MCV, Anti-Mutated Citrullinated Vimentin; CRP, C-Reactive Protein; ESR, Erythrocyte Sedimentation Rate; RF, Rheumatoid Factor.
